# Comparison between Two Decades of Prevalence of Intestinal Parasitic Diseases and Risk Factors in a Brazilian Urban Centre

**DOI:** 10.1155/2015/546705

**Published:** 2015-11-25

**Authors:** Maria Aparecida Alves de Oliveira Serra, Cristina de Souza Chaves, Zirlane Castelo Branco Coêlho, Naya Lúcia de Castro Rodrigues, Josias Martins Vale, Maria Jânia Teixeira, Francisco Josemar Alves de Oliveira, Márcio Flávio Moura de Araújo, Ivo Castelo Branco Coelho

**Affiliations:** ^1^Federal University of Ceará, Rua Monsenhor Furtado 177, Rodolfo Teófilo, 60430-350 Fortaleza, CE, Brazil; ^2^University of International Integration of the Afro-Brazilian Lusophony, Brazil

## Abstract

*Objectives*. This study's objective was to compare the prevalence of intestinal parasites and associated risk factors in children in urban communities, in the Brazilian Northeast, between two decades.* Methods*. This quantitative transversal study consisted of a comparative analysis of two different samples: the first viewing the years 1992–1996 and the other through a coproepidemiological data survey undertaken in 2010-2011.* Results*. It was evidenced that there was a reduction of intestinal parasites and that there were improvements in the socioenvironmental conditions between the two decades evaluated. It was observed that, in the period 1992–1996, playing out in the streets was associated with a higher risk for acquiring intestinal parasites. Over the 2010-2011 period, the characteristics of more than five residents per household, houses with dirt floors, children who live in homes without piped water, and children who play out in the streets were associated with a higher risk of intestinal parasitic infection.* Conclusion*. The study showed a reduction of intestinal parasitic diseases to 23.8% in 2010-2011 from 81.3% in 1992–1996 and improvement of the social-sanitary conditions of the population between the decades analyzed.

## 1. Introduction

It is estimated that, currently, more than 1 billion individuals worldwide are sheltering at least one species of intestinal parasite. Cases of* Ascaris lumbricoides*,* Trichuris trichiura,* and the hookworms predominate [1].

The infections caused by intestinal parasitic diseases have as their immediate determinants the fecal contamination of the environment and the absence or insufficiency of sanitation facilities, in addition to inadequate hygiene practices [2]. Although they have different mechanisms of transmission, all these parasites need environmental conditions that are propitious to the development of their life-history stages. As a result, communities that lack good sanitation infrastructure and sanitary education are more vulnerable to the dispersal of these agents in the environment in which they live [3].

Public health interventions, such as the provision of drinking water, health education activities, inspection of food hygiene, and the maintenance of functioning sewage systems are essential for the long-term control of intestinal parasitic diseases in the community. The implementation and sustainability of these interventions, however, are complex and vary in accordance with the local contexts [4].

In various developing countries, urban migration led to the creation of precarious urban settlements, creating a growth process of shantytowns, also termed* favelas*, on the outskirts of major urban centers, with poor sanitation conditions and high rates of intestinal parasites [5]. Shantytowns are characterized by poor housing conditions with narrow streets that are not paved; they also lack sanitation, clean water supply, and electricity as well as health services. Furthermore, the presence of high levels of crime and violence simultaneously is also common. In Fortaleza, in the state of Ceará, in the Brazilian Northeast, the population migration from the state's rural regions to the capital in the early 1990s, due to droughts and survival difficulties, promoted the growth process of* favelas* on the outskirts of the city, worsening the lack of basic sanitation, and entailing greater harm to public health and environmental degradation.

Studies on urban ecology have emphasized that population agglomerations, in conjunction with the inadequacy of basic sanitation, are risk factors for an increase in intestinal parasitic diseases in urban communities. The fecal transmission of the parasitic diseases agents can be significantly reduced through sanitation interventions in the environment, which provide an appropriate destination for human waste [6].

In the state of Ceará, Brazil, an important sanitation program was undertaken in the 1990s, increasing the sewage network infrastructure (the Fortaleza Sewage Infrastructure Program-SANEAR) to 64% from approximately 17% of the sewage in the state. The recognition of this impact and its associations will allow the development of concrete and contextualized actions with the population studied; it will, therefore, have strong implications in the implementation of preventive measures and appropriate conducts in the control of these infections.

This study's objective was to compare the prevalence of intestinal parasites and associated risk factors in children in urban communities, in Fortaleza, Brazil, between two decades. The study is also interested in determining which risk factors are associated with the presence of intestinal parasites in the two periods.

## 2. Material and Method

### 2.1. Ethical Considerations

This study was approved by the Research Ethics Committee of the Federal University of Ceará (Protocol number 60/10). In addition, after an explanation regarding the importance of the study, terms of informed consent were obtained from those responsible for the children, and the confidentiality of all the participants was assured. Children who had intestinal parasites were referred for treatment with specific antiparasitic drugs for each type of infection.

### 2.2. Area, Population, and Study Design

The study was conducted in two urban communities, Parque Universitário and Panamericano, located in the city of Fortaleza, in the Brazilian state of Ceará. The city of Fortaleza has an estimated population of 2,431,415 inhabitants. Panamericano is a neighborhood with approximately 1,520 children aged between one and twelve years, while Parque Universitário has 11,018 inhabitants and 9,197 children aged between one and twelve years [7].

This quantitative transversal study consisted of a comparative analysis of two different samples: the first viewing the years 1992–1996, and the other through a coproepidemiological data survey undertaken in 2010-2011.

In the two periods analyzed, similar criteria of eligibility were used for the sample selection. One sample of homes with children aged between 1 and 12 years was selected randomly through a census of each community, and only one child per household was randomly chosen to participate in the investigation.

As a result, the present study's sample was divided into groups with 316 children regarding the period 1992–1996 and 370 children for 2010-2011, of both sexes, with an age range of 1 to 12.

### 2.3. Data Collection

The data were provided by those responsible for the children, through home interviews in the areas selected. The demographic, socioeconomic, environmental, and sanitary information was collected by properly trained field researchers, through semistructured questionnaires handed to the children's parents or guardians. All the questionnaires were checked in relation to accuracy and perfection.

The children were asked to provide a sample of feces for a parasitological examination. Collection pots with 10% formalin, appropriately identified, were left with those responsible for the children, with instructions for the collection and conservation of the fecal matter. The feces collection instructions were the following: do not use laxatives for the feces collection; the feces shall not be mixed with urine or water and shall not be collected directly from the toilet bowl either; place one portion in the same pot during three alternate days; do not fill completely the pot; it is not necessary to keep it in the refrigerator.

The researchers collected the pots with the children's feces on two, prearranged, days of the week. The material was taken to the Parasitology Laboratory of the Department of Parasitology and Forensic Medicine of the Federal University of Ceará, where the samples were examined, processed, and subjected to the undertaking of two qualitative techniques.

Part of the sample was processed through direct method with fresh feces and direct method by using Lugol's iodine. The first consists of placing 2 to 3 drops of saline at 0.9% on a clean slide and dilute a small portion of the feces obtained from several points of the fresh feces with a wooden applicator, so that it becomes homogeneous and transparent; subsequently it is examined under optical microscope at 10x and 40x. In direct examination with Lugol's iodine staining, the examination is prepared similarly to the previous one replacing saline by Lugol's iodine [8].

The remaining part of the sample was undertaken using the spontaneous sedimentation technique (Hoffman, Pons, and Janer) [9] in 2 or 3 slides. Each replicate of conserved feces was filtered through gauze folded twice and a 125 mL polystyrene conical cup received the filtrate. Tap water was added to the filtrate up to a volume of 3/4 of the cup. The suspension was allowed to stand for 2 h, after this period, part of the sediment was collected with a pipette, and one drop was placed on a slide and stained with Lugol for subsequent microscopic examination [8]. The result was considered positive if eggs or larvae (for the helminths) or cysts (for protozoa) were found by one or the other method. Two trained laboratory technicians undertook the examination using optical microscopes with objectives of 10x and 40x.

### 2.4. Statistical Analysis

The data were entered and analyzed using the statistical software SPSS, version 22.0. Measurements of central tendency and dispersion were calculated. In all the cases, “*p*” values inferior to 0.05 were considered statistically significant. Initially, the association between the presence of intestinal parasites and risk factors and the two periods was evaluated using the Chi-squared test. The odds ratio was calculated in order to measure the strength of association. In order to determine the independent risk factors for the periods and for infection, logistic regression analysis was used.

## 3. Results

A total of 686 children was evaluated, with the male sex predominating (53.1%). Age varied between 1 and 12 years, with a mean age of 5.85 (standard deviation of 3.21). The sample was divided into groups; 316 children were from the period 1992–1996, and 370 were from the period 2010-2011. There was no association between sex and age and the groups evaluated.

### 3.1. Variation in the Prevalence of the Intestinal Parasites

The intestinal parasites were present in 345 (50.3%) of the children evaluated, with greater prevalence of* Ascaris lumbricoides*, followed by* Trichuris trichiura*,* Giardia duodenalis,* and* Entamoeba coli*, with 236 (34.4%), 188 (27.4%), 84 (12.2%), and 119 (17.3%), respectively. The prevalence of children with parasites in the period 1992–1996 was 257 (81.3%) and 88 (23.8%) in 2010-2011.

The presence of the protozoa* Giardia duodenalis* (OR 0.16, 95% CI 0.93–0.29)*, Entamoeba histolytica/dispar* (OR 0.02, 95% CI 0.004–0.19)*, Entamoeba coli* (OR 0.06, 95% CI 0.03–0.12), and* Endolimax nana* (OR 0.12, 95% CI 0.04–0.37) was associated with a lower chance of occurring in the period 2010-2011. The presence of the helminths* Ascaris lumbricoides* (OR 0.10, 95% CI 0.07–0.15)*, Trichuris trichiura* (OR 0.11, 95% CI 0.07–0.16), and Ancylostomatidae (OR 0.03, 95% CI 0.005–0.28) was associated with a lower chance of occurrence in the period 2010-2011, in the uni- and multivariate analyses (Table 1).

### 3.2. Variation in the Risk Factors

Factors such as the presence of more than five persons per household (OR 0.31, 95% CI 0.22–0.42), dirt floor (OR 0.14, 95% CI 0.08–0.24), not having piped water (OR 0.05, 95% CI 0.02–0.10), houses with less than four rooms (OR 0.28, 95% CI 0.19–0.40), untreated drinking water (OR 0.71, 95% CI 0.52–0.97), lack of public sewage network (OR 0.12, 95% CI 0.005–0.27), and lack of garbage collection (OR 0.21, 95% CI 0.11–0.38) were associated with a lower chance of occurrence in the period 2010-2011 (Table 2).

Houses without a toilet (OR 33.7, 95% CI 8.14–140) and children who did not have the habit of washing their hands (OR 13.7, 95% CI 7.80–24.1) and who went barefoot (OR 5.24, 95% CI 1.48–18.5) were associated with a higher chance of occurrence in the period 1992–1996. Children who played out in the street (OR 0.50, 95% CI 0.36–0.69) and made use of antiparasitic medication (OR 0.57, 95% CI 0.41–0.79) were associated with a lower chance of occurrence in the period 2010-2011. In the multivariate analysis, the variables of presence of toilets and children who played out in the street lost significance (Table 2).

### 3.3. Intestinal Parasites and Risk Factors

When the two periods were evaluated separately in relation to the occurrence of intestinal parasites, based on socioenvironmental variables, it was observed that, in the period 1992–1996, playing out in the street was associated with a higher risk for acquiring intestinal parasites (OR = 1.83, 95% CI = 1.006–3.33) in the univariate analyses. However, no variable was associated with the presence of intestinal parasitic disease in the multivariate analyses (Table 3).

In the period 2010-2011, the characteristics of more than five residents per household (OR = 2.11, 95% CI = 1.27–3.52), houses with dirt floor (OR = 4.33, 95% CI = 1.73–10.8), children living in homes without piped water (OR = 5.08, 95% CI 1.40–18.4), and children that play out in the street (OR = 2.52, 95% CI 1.48–4.28, *p* = 0.001) were associated with a higher risk of intestinal parasitic infection (Table 3). In the multivariate analysis, children with a higher number of people in the family (*p* = 0.05), houses with earth floors (*p* = 0.04), and children who play in the street (*p* = 0.006) maintained statistical association with parasitic infection.

## 4. Discussion

The present study sought to clarify the changes that occurred in two decades in the prevalence of intestinal parasitic diseases and in the sociosanitary scenario of two urban communities of Fortaleza, Ceará, Brazil. It was evidenced that there was a reduction of intestinal parasites and that there were improvements in the socioenvironmental conditions between the two decades evaluated.

All the species of intestinal protozoa analyzed became less frequent in the period of 2010. The pathogenic protozoan* Giardia duodenalis* and the nonpathogenic* Entamoeba coli* were the most prevalent in the two periods and underwent a significant reduction between the decades analyzed. Studies undertaken in the southern region of Brazil also demonstrated a reduction in the prevalence of these protozoa and were associated with the improvement of the community's sanitation conditions [10, 11].

Water is an important route of transmission of* Giardia duodenalis*, either by direct ingestion of cysts or indirectly by consumption of food and beverage prepared with contaminated water. Furthermore, accidental contamination can occur during recreational activities. Besides water transmission, direct transmission from person to person is common among children through contaminated hands and in crowded places [12, 13]. Over the 1992–1996 period, there were poor sanitation conditions, water treatment, and hygiene habits which may explain a higher protozoan parasite contamination in the sample studied during this period. In the period 2010-2011, there was a significant improvement in the sanitation conditions and water treatment; however, as* Giardia duodenalis* persisted it may suggest the direct transmission of cysts among children facilitated by crowded families, which was associated with the presence of parasites in this period.

In the present study, the prevalence of geohelminths (*A. lumbricoides, T. trichiura,* and Ancylostomatidae) reduced significantly, similarly to the findings in Salvador (Bahia, Brazil) [2] and in Caxias do Sul (Rio Grande do Sul, Brazil) [14].* A. lumbricoides* and* T. trichiura* were the most prevalent geohelminths in the two periods; these parasites use similar transmission strategies, and a large number of highly resistant eggs are liberated through the feces into the environment, remaining viable for long periods. Even under ideal hygiene conditions, the environment can remain contaminated with the eggs, ensuring continuity of transmission [15].

Geohelminths need the soil to complete their biological cycle. This information is fundamental to understand the epidemiology and clinical and control actions of the infections caused by these helminths. These parasites are transmitted to human hosts through the ingestion of mature eggs or skin penetration of infective larvae developed in moist soil contaminated with human waste [16, 17].

Children with inadequate hygiene habits such as not using the toilet and not washing hands after defecation enable the geohelminths transmission through the subungual deposition of viable eggs. This, added to the habit of bringing the hands to the mouth, favors the fecal-oral transmission of geohelminths such as* Ascaris lumbricoides* and* Trichuris trichiura.* Besides, habits like not wearing shoes increase the probability of geohelminth transmission such as* Necator americanus, Ancylostoma duodenale,* and* Strongyloides stercoralis*, whose transmission route is the penetration of infective larvae in the skin, considering that the two latter ones can also be transmitted through the ingestion of infective larvae [17, 18].

Studies showed that washing hands after defecation and wearing shoes reduce the risk of acquiring geohelminths [19, 20]. The present study showed that homes without toilet facilities and children that did not have the habit of washing hands after defecation and wearing shoes were more frequent over the period 1992–1996, a fact that may explain a higher prevalence of geohelminths in this period.

In the period 1992–1996, it was observed that children's habit of playing out in the street was associated with the acquisition of intestinal parasites. This fact suggests that children in contact with a contaminated environment were vulnerable in relation to geohelminths such as* A. lumbricoides* and* T. trichiura,* as their transmission needs soil for maturation of their life-history forms. Another factor influencing the high prevalence of these geohelminths in the above-mentioned period is the low access to the public sewage network (98% did not have this service), with difficulty accessing a sewer system being an extremely important factor in environmental contamination.

In one analysis of municipalities with low human development indices in the North and Northeast of Brazil, Fonseca and collaborators [21] found that crowded family and presence of human waste and garbage near the house were risk factors for the acquisition of intestinal parasites. Similarly, Barreto and collaborators [2, 4, 22] evaluated various aspects of the program for constructing sewage systems in Salvador (Bahia, Brazil) and found that basic sanitation performed an essential role in promoting public health, leading to a reduction in the prevalence of intestinal parasitic infection caused by geohelminths, as it reduced environmental contamination.

The SANEAR public program was extended in these communities at the end of the 1990s; after 15 years, clear effects of the program were observed in the improvement of the health conditions of the communities analyzed. One must take into account, however, that other factors already described also contributed to this, such as the reduction in the size of families, improvement in housing and hygiene habits, and use of antiparasitic drugs.

Studies have demonstrated that the use of treated drinking water [23, 24] and access to piped water [25, 26] as well as an appropriate destination for human waste [20, 25] are associated with the reduction of the geohelminthiases. The present study showed a significant improvement between the two decades, of the water provided to the communities and of the human waste destination, through the expansion of SANEAR program.

During the research, there was a reduction of the prevalence of intestinal parasites, possibly related to issues like improvement in sanitation conditions and hygiene habits such as washing hands and wearing shoes. However, factors like crowded families, unpaved floor, and recreational activities in contaminated environments allow the persistence of intestinal parasites transmission in this community. Such points favor environmental contamination and justify the need of expanding the sanitation and economic infrastructure in order to mitigate these factors.

The present study has some methodological limitations. Initially, there was no monitoring of the children in the study, and only one stool sample was collected per child. As the collection period was short, possible seasonal fluctuations may have affected the real prevalence. Due to the lack of antigen tests,* Entamoeba histolytica* and* Entamoeba dispar* were not separated. Parasitological quantitative fecal examination techniques were not used for the parasite burden evaluation. Finally, the laboratory techniques used have low sensitivity for detecting some intestinal parasites, such as* Strongyloides stercoralis* and* Enterobius vermicularis.* The first is due to the low density of parasites and minimal and irregular release of larvae in the feces, and the second is due to the biological behavior of this helminth, whose pregnant females actively migrate to the host's perianal region where they leave their eggs adhered and do not need the feces to transport their eggs [17].

In spite of the limitations, the present study's results show that changes in the urban socioenvironmental context influence the population's health, reducing the prevalence of infections caused by intestinal parasites.

## 5. Conclusion

The study showed a reduction from 81.3% in 1992–1996 to 23.8% in 2010-2011 of intestinal parasitic diseases, and improvement of the sociosanitary conditions of the population between the decades analyzed.

The protozoan* Giardia duodenalis* and the geohelminths* A. lumbricoides* and* T. trichiura,* in spite of the significant reduction between the periods evaluated, continue to parasite the communities' children.

There is currently a rise in cases of chronic noncommunicable diseases in Brazil and worldwide; however, the investigation of problems such as diseases caused by parasites must not be neglected. Epidemiological studies of this type are important, as, indirectly, they describe the access to, and quality of, basic issues for promotion of human health, such as washing, care with food, potable water, and a sewage network.

As a result, the undertaking of similar studies is important for constructing the current panorama of parasitic disease and promoting the eradication of this public health problem.

## Figures and Tables

**Table 1:** Prevalence of intestinal parasites in stool collected in the period 1992–1996 and in 2010, Northeast Brazil.

Intestinal parasites	Period		Univariate analysis			Multivariate analysis		
	Period: 1992–96 *N* = 316 *n* (%)	Period: 2010 *N* = 370 *n* (%)	*p*	OR	95% CI	*p*	OR	95% CI
Protozoa								
* Giardia duodenalis*	68 (21.5)	16 (4.3)	<0.001	0.16	0.09–0.29	<0.001	0.26	0.13–0.51
* Entamoeba histolytica/dispar*	29 (9.2)	01 (0.3)	<0.001	0.02	0.004–0.19	0.05	0.12	0.01–1.08
* Entamoeba coli*	107 (33.8)	12 (3.8)	<0.001	0.06	0.03–0.12	<0.001	0.13	0.06–0.26
* Endolimax nana*	25 (7.9)	04 (1.3)	<0.001	0.12	0.04–0.37	0.02	0.23	0.06–0.83
Helminthes								
* Ascaris lumbricoides*	187 (59.2)	49 (15.5)	<0.001	0.10	0.07–0.15	<0.001	0.19	0.12–0.31
* Trichuris trichiura*	153 (48.4)	35 (11.1)	<0.001	0.11	0.07–0.16	0.001	0.42	0.24–0.71
Ancylostomatidae	21 (6.6)	01 (0.3)	<0.001	0.03	0.005–0.28	0.04	0.10	0.01–0.93
* Enterobius vermicularis*	13 (4.1)	05 (1.6)	0.02	0.31	0.11–0.90	0.85	0.89	0.26–2.97
* Hymenolepis nana*	21 (6.6)	02 (0.6)	<0.001	0.07	0.01–0.32	0.04	0.17	0.03–0.92

**Table 2:** Prevalence of potential risk factors collected in the period 1992–1996 and in 2010, Northeast Brazil.

Risk factors	Period		Univariate analysis			Multivariate analysis		
	1992–96 *N* = 316 *n* (%)	2010 *N* = 370 *n* (%)	*p*	OR	95% CI	*p*	OR	95% CI
Persons per household								
≤5	142 (44.9)	268 (72.4)	<0.001	0.31	0.22–0.42	<0.001	0.28	0.17–0.46
>5	174 (55.1)	102 (27.6)						
Floors conditions								
Ceramic/cement	228 (72.6)	350 (94.3)	<0.001	0.14	0.08–0.24	0.02	0.33	0.13–0.84
Stamped earth floor	88 (27.8)	20 (5.4)						
Piped water inside the household								
Yes	210 (66.5)	360 (97.3)	<0.001	0.05	0.02–0.10	<0.001	0.18	0.07–0.46
No	106 (33.5)	10 (2.7)						
Rooms of the house								
>4	59 (18.7)	166 (44.9)	<0.001	0.28	0.19–0.40	<0.001	0.34	0.20–0.58
≤4	257 (81.3)	204 (55.1)						
Toilets								
Yes	267 (84.5)	368 (99.5)	<0.001	33.7	8.14–140	0.09	3.86	0.80–18.5
No	49 (15.5)	02 (0.50)						
Drinking water treatment								
Yes	197 (62.3)	201 (55.3)	0.03	0.71	0.52–0.97	<0.001	0.26	0.15–0.44
No	119 (37.7)	169 (45.7)						
Excreta disposal								
Yes	06 (1.9)	230 (62.2)	<0.001	0.12	0.005–0.27	<0.001	0.14	0.005–0.03
No	310 (98.1)	140 (37.8)						
Garbage collection								
Yes	263 (83.2)	355 (95.9)	<0.001	0.21	0.11–0.38	0.04	0.41	0.17–0.96
No	53 (16.8)	15 (4.1)						
Hands washing habit								
Yes	200 (63.3)	355 (95.9)	<0.001	13.7	7.80–24.1	<0.001	4.92	2.19–11.0
No	116 (36.7)	15 (4.1)						
Wearing shoes habit								
Yes	303 (95.9)	367 (99.2)	0.004	5.24	1.48–18.5	0.01	21.6	2.11–222.5
No	13 (4.1)	03 (0.8)						
Children playing out the street								
Yes	231 (73.1)	214 (57.8)	<0.001	0.50	0.36–0.69	0.41	0.80	0.47–1.36
No	85 (26.9)	156 (42.2)						
Use of antiparasitic medication								
Yes	231 (73.1)	226 (61.1)	0.001	0.57	0.41–0.79	0.05	0.58	0.33–1.009
No	85 (26.9)	144 (38.9)						

**Table 3:** Univariate analyses of intestinal parasitic infections and potential risk factors in the period 1992–1996 and in 2010, Northwest Brazil.

Risk factors	Parasite positive	Period			Parasite positive	Period		
	*N* = 257	1992–1996			*N* = 88	2010		
	*n* (%)	*p*	OR	95% CI	*n* (%)	*p*	OR	95% CI
Persons per household								
≤5	111 (43.2)	0.19	1.45	0.82–2.56	53 (60.2)	0.003	2.11	1.27–3.52
>5	146 (56.8)				35 (39.8)			
Floors conditions								
Ceramic/cement	186 (72.4)	0.85	0.94	0.50–1.76	77 (87.5)	0.001	4.33	1.73–10.8
Dirt floor	71 (27.6)				11 (12.5)			
Piped water inside the household								
Yes	168 (65.4)	0.39	1.30	0.70–2.43	82 (93.2)	0.006	5.08	1.40–18.4
No	89 (34.6)				06 (6.8)			
Rooms of the house								
>4	48 (18.7)	0.99	0.99	0.48–2.06	33 (37.5)	0.11	1.48	0.91–2.43
≤4	209 (81.3)				55 (62.5)			
Toilets								
Yes	220 (85.6)	0.25	1.51	0.73–3.12	87 (98.9)	0.38	0.31	0.19–5.00
No	37 (14.4)				01 (1.1)			
Drinking water treatment								
Yes	161 (62.6)	0.81	1.07	0.59–1.91	40 (45.5)	0.05	0.62	0.38–1.01
No	96 (37.4)				48 (54.5)			
Excreta disposal								
Yes	05 (1.9)	0.89	0.86	0.10–7.58	49 (55.7)	0.15	1.42	0.87–2.31
No	252 (98.1)				39 (44.3)			
Garbage collection								
Yes	212 (82.5)	0.46	1.35	0.60–3.04	85 (96.6)	0.72	0.79	0.21–2.88
No	45 (17.5)				03 (3.4)			
Hands washing habit								
Yes	167 (65.0)	0.19	1.46	0.82–2.59	39 (44.3)	0.81	0.94	0.58–1.52
No	90 (35.0)				49 (55.7)			
Wearing shoes habit								
Yes	247 (96.1)	0.67	1.32	0.35–4.96	87 (98.9)	0.69	0.62	0.05–6.93
No	10 (3.9)				01 (1.1)			
Children playing the street								
Yes	194 (75.5)	0.04	1.83	1.00–3.33	65 (73.9)	<0.001	2.52	1.48–4.28
No	63 (24.5)				23 (26.1)			
Use of antiparasitic medication								
Yes	186 (72.4)	0.54	0.81	0.42–1.57	49 (55.7)	0.35	0.79	0.49–1.28
No	71 (27.6)				39 (44.3)			
